# Environmental and Anthropogenic Impacts on Avifaunal Assemblages in an Urban Parkland, 1976 to 2007

**DOI:** 10.3390/ani4010119

**Published:** 2014-03-17

**Authors:** Sara Elizabeth Ormond, Robert Whatmough, Irene Lena Hudson, Christopher Brian Daniels

**Affiliations:** 1 School of Natural and Built Environments, University of South Australia, Mawson Lakes Campus, P.O. Box 2471, Adelaide, SA 5000, Australia; 2 11 Wakefield, St Kent Town, SA 5067, Australia; E-Mail: bobwhatmough@bigpond.com; 3 School of Mathematics and Statistics, University of South Australia, Mawson Lakes Campus, P.O. Box 2471, Adelaide, SA 5000, Australia; 4 Barbara Hardy Institute, University of South Australia, Mawson Lakes Campus, P.O. Box 2471, Adelaide, SA 5000, Australia

**Keywords:** birds time series, urban parklands abundance diversity

## Abstract

**Simple Summary:**

Over 32 years, the bird species assemblage in the parklands of Adelaide showed a uniform decline. Surprisingly, both introduced and native species declined, suggesting that even urban exploiters are affected by changes in the structure of cities. Climate and anthropogenic factors also cause short term changes in the species mix. In the case of Adelaide, the drought of 2005–2007 and associated water restrictions profoundly impacted the avian assemblage using the city parklands.

**Abstract:**

Urban environments are unique, rapidly changing habitats in which almost half of the world’s human population resides. The effects of urbanisation, such as habitat (vegetation) removal, pollution and modification of natural areas, commonly cause biodiversity loss. Long-term ecological monitoring of urban environments is vital to determine the composition and long-term trends of faunal communities. This paper provides a detailed view of long-term changes in avifaunal assemblages of the Adelaide City parklands and discusses the anthropogenic and environmental factors that contributed to the changes between 1976 and 2007. The Adelaide City parklands (ACP) comprise 760 ha of land surrounding Adelaide’s central business district. Naturalist Robert Whatmough completed a 32-year survey of the ACP to determine the structure of the urban bird community residing there. Annual species richness and the abundance of birds in March and September months were analysed. Linear regression analysis was applied to species richness and abundance data of each assemblage. Resident parkland birds demonstrated significant declines in abundance. Native and introduced species also exhibited long-term declines in species richness and abundance throughout the 32-year period. Cycles of varying time periods indicated fluctuations in avian biodiversity demonstrating the need for future monitoring and statistical analyses on bird communities in the Adelaide City parklands.

## 1. Introduction

Urbanisation is described as the progression of anthropogenic disturbance and human construction to create communities) [1]. Land fragmentation and particularly habitat loss, caused by urbanisation, has been the source of global biodiversity depletion spiking an increase in ecological studies of urban environments [2,3,4,5,6,7]. Urban environments differ from “natural” environments because maximum temperatures are generally higher than average and monthly rainfall is relatively variable [8]. Urban environments are also usually situated on flat, heavily paved surfaces and generate high levels of pollution that can cause an urban heat island (UHI) effect consequently increasing stress on urban wildlife [9,10]. However, most importantly urban environments replace indigenous habitat with a mixture of introduced and native plants, new soils, altered water regimes and with a completely different structure and complexity. For these reasons urban environments are commonly studied as unique habitats containing faunal assemblages that differ from those in “natural” areas [11].

Of the increasing studies conducted on urban ecology, birds are often monitored because of their visibility, cost effectiveness for surveying and ability to act as indicators for ecological health [11]. Bird communities in urban environments are unique because they commonly contain low species richness with relatively high abundances [5]. The abnormal structure of urban bird communities is said to be attributed to birds having different adaptability thresholds where some birds avoid urban environments and some rely on them for survival [11]. The latter types of birds are referred to as “urban exploiters” or “urban matrix occupiers” that have readily adapted to urban environments enabling their abundance to skyrocket and subsequently causing low community diversity [12]. 

Previous studies conducted along suburban to highly urban gradients indicated reduced biodiversity within highly urban areas [1,13,14]. A majority of urban bird studies have previously been conducted in northern hemisphere environments which has created a paucity of information on southern hemisphere bird communities [15]. The high proportion of nectivorous birds in Australian environments [16] as well as unique vegetation structures and climatic cycles hinder comparison of urban bird studies in northern and southern hemisphere environments [5].

Temporal studies in urban environments are conducted infrequently, particularly in Australia. The lack of historical biodiversity documentation in urban settings creates difficulty when determining the effects of anthropogenic disturbances [12]. Urban-induced pressures that can affect faunal inhabitants include high density of human populations, domestic animal predation, transport networks, pollution, UHI effect, habitat degradation and fragmentation and changes in land use [12]. The aforementioned effects of urbanisation are usually detrimental to flora and fauna, yet few ecological studies are conducted to inform management strategies in urban environments that could potentially reduce the adverse effects of urbanisation [17]. Long-term ecological monitoring is vital to satisfy gaps in knowledge regarding changes in urban biodiversity [5] and the effects of urbanization [12].

This paper provides a detailed view of long-term changes in avifaunal assemblages of the Adelaide City parklands. Specific questions answered are: (i) What long-term changes have occurred within avifaunal assemblages from 1976 to 2007? and (ii) The anthropogenic and environmental factors that could cause the observed changes to avifaunal assemblages is discussed

## 2. Methods

### 2.1. Study Site

The Adelaide City parklands (ACP) comprise 45% of Adelaide City and have endured extensive vegetation clearance and habitat fragmentation since its original composition of diverse, native vegetation (Adelaide City Council 2008a). The ACP comprise a 760 Ha ring of open woodland, sports fields, formal gardens and indigenous plantings that surrounds the city center. The parklands are relatively free of built structures, although there are a few small buildings for sporting clubs in the Southern Parklands. The Western Parklands is also the location for a large High School. The ACP currently comprises a predominately open woodland habitat containing native and introduced vegetation. The open woodland environment largely contains scattered old-growth trees and lacks understory vegetation [6,9]. Native trees such as Eucayptus species (*Eucalyptus* *camaldulensis* and *Corymbia citriodora*) and fig trees (*Ficus rubiginosa and Ficus macrophylla*) are common in the parklands along with introduced species such as *Jacaranda mimosifolia,* *Olea europaea*, and plane tree species (*Platanus acerfolia* and *Platanus orientalis*) [18,19]. Adelaide City has a Mediterranean climate (Australian Bureau of Statistics 2008) with relatively mild winters (the average long-term maximum is 16.8 °C) and hot dry summers (the average long-term maximum is 27.73 °C) [20]. The average rainfall in Adelaide City is 504.44 mm per annum (from 1976 to 2006). Drought conditions in the last three years of the data collection period have caused a slightly lower average rainfall of 479.73 mm (from 2000 to 2006) [21].

### 2.2. Bird Surveys

**Figure 1:**
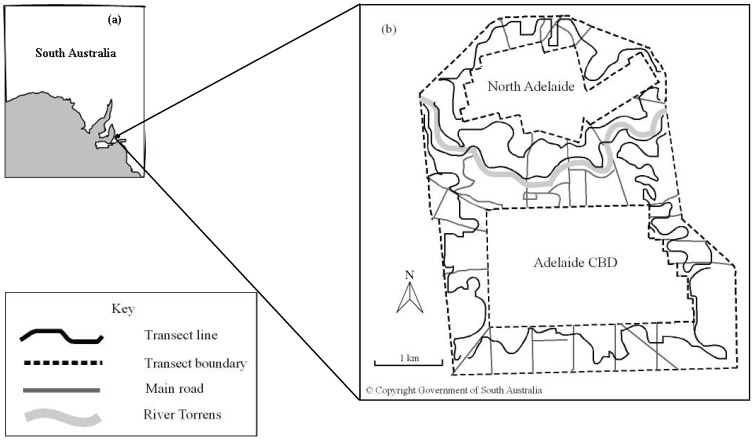
(**a**) Map of South Australia indicating Adelaide City [22]. (**b**) Bird survey transects in the Adelaide City parklands [23].

Surveys were conducted from 1974 to 2008 by naturalist, Robert Whatmough. He used six transect lines that allowed a majority of Adelaide’s urban parklands to be covered (Figure 1). Each transect is approximately six km long and covered: north Adelaide, central Adelaide, the River Torrens, west Adelaide, south Adelaide and east Adelaide. Each month he walked transects and recorded bird species and number of individuals, either seen or heard within transect boundaries. Large flocks of birds were identified and estimated in numbers. Three transects were surveyed per day, thus two days of surveying occurred each month. The north Adelaide, central Adelaide and east Adelaide transects were completed in one survey day. The River Torrens, west Adelaide and south Adelaide transects were completed in another survey day. Travel along transects were alternated between ascending and descending order each month, to remove any possible bias caused by the time of day sampling activities were conducted. Each set of three surveys were conducted on weekends when bird calls were less masked by traffic noise. Survey days were selected according to favourable weather forecasts of mild weather. Undesirable weather conditions (such as rain or extreme heat) were avoided. However, once a survey had commenced, it would continue regardless of weather conditions. Each survey day usually commenced between 8:00 am and 10:00 am and required approximately five hours to complete three transects. Robert Whatmough commenced this monitoring in 1974 and is currently still surveying bird communities in the Adelaide city parklands. Calendar years 1976 to 2007 were extracted for this study.

### 2.3. Statistical Analysis

Change Point Analyser (CPA), or cumulative sum analysis (CUSUM) change point analysis, was used to examine periods of abrupt change within the time series. CPA is used to complement time series analysis by identifying and generating confidence bands for period of rapid change [24]. Linear regression analysis was used to plot lines of best fit.

## 3. Results

**Figure 2:**
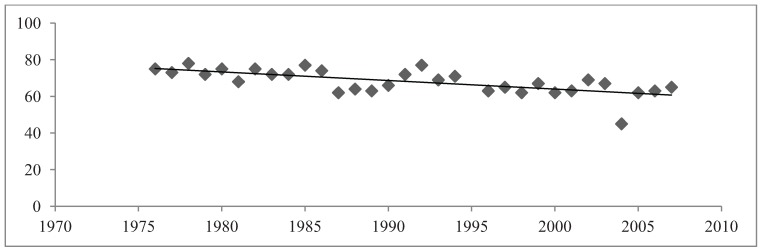
Total Species Richness in March and September months (combined).

The avian assemblage declined over the 32 year period and, especially declined in years 2005 to 2007 (Figure 2). The measure of species richness was used as a diversity index in order to outline the long-term changes in species richness of Adelaide parkland birds. In the Adelaide City parklands from the years 1976 to 2007 the diversity of avifaunal species richness fluctuated from 45 species to 78 species (Figure 2). The mean species richness of birds was 68 (±1.209) species. 1978 saw the highest species richness with 78 species present in the parklands (Figure 2). The lowest measure of species richness was recorded in 2004 revealing only 45 recorded species. Linear regression analysis indicated a highly significant, negative relationship between species richness and the 32-year study period (R^2^ = 0.439, Sig. ≤ 0.001). Overall the species richness of birds recorded in the Adelaide City parklands shared a highly significant, strong, negative relationship with the 32-year time period. 

One hundred and thirty-four different bird species were recorded in the Adelaide City parklands from 1976 to 2007. Forty-five bird species were classified as resident species. Resident species were, unsurprisingly, present in the parklands virtually every year and comprised 33.6% of species recorded in the parklands within the 32-year study period. Annually, a mean number of 68 (±standard error 1.208) species were recorded in the parklands each year. Resident birds had a mean number of 45 (±0.201) species present each year and comprised a majority of the annual species richness. Twelve commonly recorded species were seen in the parklands throughout the 32-year study period with an annual mean species richness of 9 (±0.3) species. Commonly recorded birds comprised 9% of the species richness of parkland birds. The occasionally recorded species comprised 12.7% of bird species in the parklands. Seventeen bird species were classified as occasionally recorded species and comprised a mean number of 8 (±0.417) species present in each study year. Sixty species were classified as rarely recorded species and comprised 44.8% of bird species recorded in the parklands. However, 31 of the rare species were present in only one or two study years. A mean number of 7 (±0.614) rarely recorded species were present in the parklands each year indicating that only a small proportion of these birds are seen in the parklands annually. Commonly and occasionally recorded species were recorded in relatively small proportions. Resident species encompassed approximately one third of the 134 recorded species. Rarely recorded birds contained the largest percentage of birds recorded in the parklands throughout the 32-year study period; however only a small proportion was present in each study year.

**Figure 3:**
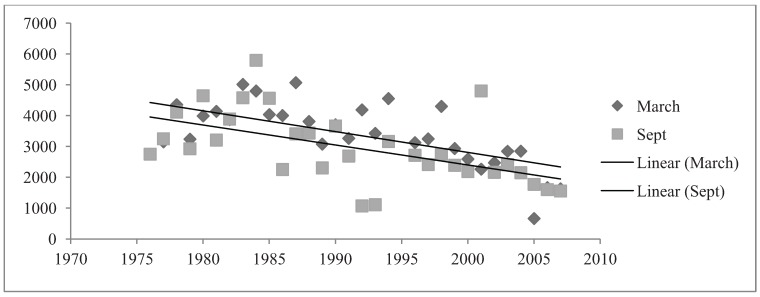
Total Species Abundance in March and September months.

Abundance data were examined by comparing the number of birds recorded in March and September from 1976 to 2007 (Figure 3). In both March and September the resident species comprised a large majority of bird abundance (Figure 3). Overall, March and September months were similar in mean abundance calculations (Figure 3). 

**Figure 4:**
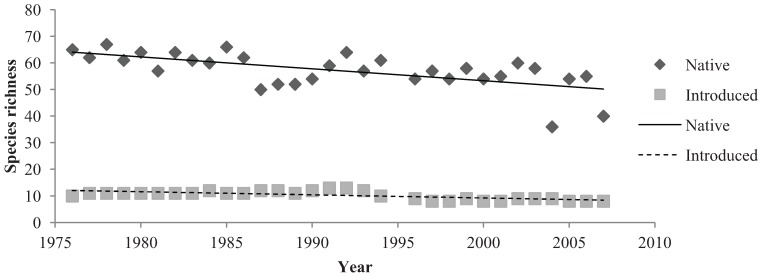
The annual species richness of native and introduced birds in the Adelaide City parklands from 1976 to 2007.

**Figure 5:**
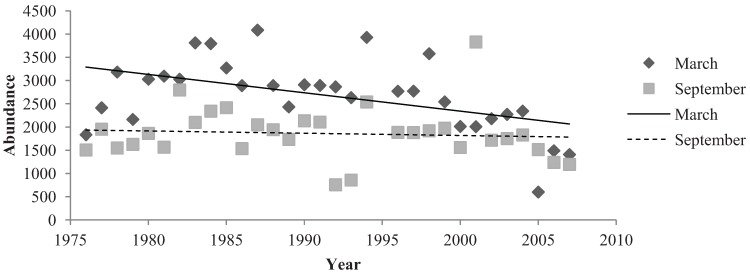
The abundance of native birds in March and September months in the Adelaide City parklands from 1976 to 2007.

**Figure 6:**
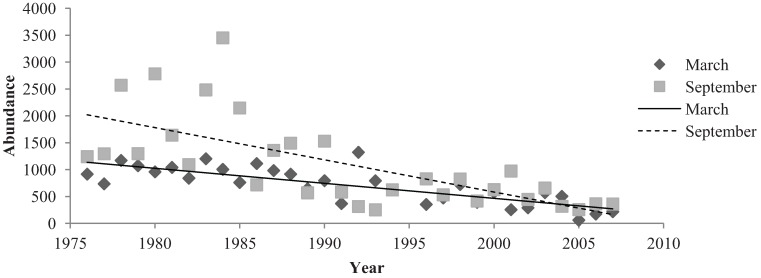
The abundance of introduced birds in March and September months in the Adelaide City parklands from 1976 to 2007.

Linear regression analysis was performed on species richness data of native and introduced birds to determine long-term changes in native and introduced assemblages (Figure 4, Figure 5 and Figure 6). Native birds exhibited many small changes in species richness throughout the 32-year period (Figure 4 and Figure 5). Linear regression analysis indicated that native bird species in the parklands demonstrated a significant, negative relationship with the 32-year study period (R^2^ = 0.304, Sig. = 0.001). The R^2^ value indicated that this relationship was moderately strong. Linear regression analysis indicated a significant, negative relationship between introduced species in the parklands and the 32-year monitoring period (R^2^ = 0.471, Sig ≤ 0.001). The R^2^ value signified that this was a relatively strong relationship (Figure 4 and Figure 6). 

Native birds comprised a majority of the total bird abundance in both March and September months (Figure 5). The mean abundance of native birds in the parklands was 2683 (±139.249) birds in March months and 1860 (±102.631) birds in September months (Figure 5). In March, native birds demonstrated a significant, negative relationship with the 32 years of study (R^2^ = 0.235, Sig. = 0.006). Although this regression relationship was identified as significant, the R^2^ value was not particularly strong. CUSUM change point analysis of the native bird abundances in March showed a significant change in the years 1998 to 2001 (95% confidence level). The relationship of native birds and September months from 1976 to 2007 was negative but non-significant (R^2^ = 0.007, Sig. = 0.0663). Additionally, no significant changes were detected by CUSUM change point analysis. Overall native bird abundances differed in March and September months. The sign test for paired data illustrated a high level of significant (Sig. < 0.001) (Figure 5). 

Introduced bird abundances in March and September demonstrated significant, negative relationships from 1976 to 2007. Introduced birds had a mean abundance of 705 (±60.573) birds in March months and a mean number of 1099 (±149.61) birds in September months (Figure 6). A very significant, negative relationship existed between March months and the 32-year period (R^2^ = 0.003, Sig. ≤ 0.001). CUSUM change point analysis of the introduced bird abundances in March showed there was a significant change from the years 1993 to 1997 (95% confidence level). September months demonstrated a relatively strong regression relationship between introduced species abundance and the 32 years (R^2^ = 0.468, Sig. ≤ 0.001). CUSUM change point analysis for September bird abundances saw changes from 1983 to 1987 and again in the year 2005 (95% confidence level). The sign test for paired data demonstrated that introduced bird abundances in March and September months were significantly different (Sig. = 0.001) (Figure 6).

The abundances of native birds shared a significant, negative relationship with March months but did not indicate a significant relationship with September months. The abundance of introduced birds exhibited significant, negative relationships with both March and September months. March and September both contained significantly different introduced and native bird abundances which were illustrated by the sign test for paired data.

## 4. Discussion

Urban ecological studies often include introduced species because of their invasive nature and the negative impacts they can have on bird communities [25]. Of the 134 species recorded in the ACP throughout the 32-year period, 118 were native species and 16 were introduced. The relatively high proportion of introduced species is supported by previous studies that state introduced birds are adaptable to urban environments and can act as “urban exploiters” [26]. Although some species appeared in vast numbers (e.g., *Chroicocephalus novaehollandiae, Ocyphaps lophotes, Sturnus vulgaris* and *Eolophus roseicapillus*), analysis illustrated significant declines in native, introduced and all species. Bird abundance was more variable than species richness which created some peaks and troughs in the time series (in particular the total bird abundance and native bird abundance). A large majority of the abrupt changes in the time series were caused by a few dominant species rapidly changing in abundance. Abrupt changes within the native bird time series were commonly attributed to a combination of dominant bird species such as *Eolophus roseicapillus* (galah), *Chroicocephalus novaehollandiae,* (silver gull), *Hirundo neoxena* (welcome swallow), *Ocyphaps lophotes* (crested pigeon), *Manorina melanocephala* (noisy miner) and *Chenonetta jubata* (Australian wood duck). Similarly *Sturnus vulgaris* (common starling), *Columba livia* (feral rock dove), *Anas platyrhynchos* (mallard) and *Passer domesticus* (house sparrow) were primarily responsible for rapid declines and increases within the introduced bird time series. The decline of both native and introduced species throughout the time series indicates that the assemblages may have been exposed to disturbances that lead to their imminent depletion. Many potential factors could have attributed to these declines including climate, rainfall, anthropogenic interference and increased urbanisation; all of which will be reviewed within this discussion.

Changes in climate and rainfall can affect the distribution of individual bird species and composition of bird communities. Increased temperatures are usually unfavourable to Australian avifauna, influencing changes in migration patterns and mistimed reproduction [27,28,29]. Annual mean maximum temperatures in the City of Adelaide have seen a gradual increase from 1976 to 2008. March and September temperatures have also increased throughout the study period [20]. Whether this is a result of global climate change cannot be determined in this study, although it should be noted that 2006 recorded the highest mean maximum temperature (23.7 °C) of all years from 1976 to 2008 and the lowest measure of annual rainfall (287.6 mm) [21]. In contrast, 1978 and 1992 recorded the lowest mean maximum temperature of 21 °C [20].

The Adelaide City Council [9] state that the central business district (CBD) of Adelaide is subject to ‘urban heat island (UHI) effect’. UHI effect is the increase of temperature in highly urbanised areas because of dark surfaces such as paved areas or roads [8,30]. UHI data have not been collected and calculated for the City of Adelaide. Increased temperatures within the ACP, intensified by the UHI effect could influence the migration and/or survival rate of bird species. 

Urbanisation and changes in land use management are common forms of human intervention in urban environments. Urbanisation has increased in the City of Adelaide throughout the 32 year period and changes have included increases in human population densities and the construction of high-rise buildings [31,32]. Both these activities contribute to habitat loss. Urbanisation affects bird communities negatively by creating pollution, habitat loss and fragmentation and landscape degradation. Impacts of urbanisation may have particularly influenced native birds as they are less adaptable to urban environments than introduced species [26].

## 5. Conclusion

Bird assemblages in the Adelaide City parklands have demonstrated significant long-term changes from 1976 to 2007. A majority of bird species recorded in the Adelaide parklands, on an annual basis, were resident species. Twelve resident species were identified as the most dominant in the community and classified as urban exploiters. These dominant species dramatically influenced the long-term changes of assemblages. Some assemblages contained one or two dominant species that caused rapid fluctuations in the time series. Occasionally recorded birds and rarely recorded birds were thus affected by *Cacatua tenuirostris* and *Anser anser* respectively. *L. novaehollandiae, S. vulgaris* and *O. lophotes* were the three most frequently dominant species and were highly influential to the long-term changes in assemblages.

Changes in management techniques [6], climatic conditions [20], human population densities [32], and the adverse effects of urbanisation [12] were likely factors that influenced declines in bird communities. Assemblages that declined in abundance almost always demonstrated a dramatic decrease in the years 2005 to 2007. Whether this decrease is temporary is unclear indicating that future monitoring of parkland bird communities must continue.

The abundance of birds in March and September months was significantly different. March exhibited higher abundances of birds than September. Intense heat in March months may attract bird species to the Torrens River water source in the parklands [6]. The decline in species richness could be detrimental to bird communities and ecosystems within the parklands. However, underlying cycles with different time periods indicate an array of potential biotic and abiotic factors of influence.

Temporal community studies are valuable to scientific research and conservation of avifaunal communities because long-term datasets can be utilised to determine underlying patterns within communities and lead to the prediction of future trends. Understanding the composition and ecological changes within communities is merely a preliminary step in conservation. Temporal studies can be applied to a variety of analyses, demonstrated within this study. Future ecological monitoring of parkland birds communities must continue so that further declines in species richness and abundance may be remedied with suitable management techniques.
